# The use of phosphate rock and plant growth promoting microorganisms for the management of *Urochloa decumbens* (Stapf.) R.D. Webster in acidic soils

**DOI:** 10.7717/peerj.18610

**Published:** 2024-12-06

**Authors:** Alexandro Barbosa, Isbelia Reyes, Alexis Valery, Carlos Chacón Labrador, Oscar Martínez, Maximo F. Alonso

**Affiliations:** 1 Escuela de Graduados, Facultad de Ciencias Agrarias y Alimentarias, Universidad Austral de Chile, Valdivia, Los Ríos, Chile; 2 Decanato de Investigación, Universidad Nacional Experimental del Táchira, San Cristobal, Tachira, Venezuela; 3 Departamento de Ingeniería Agronómica, Universidad Nacional Experimental del Táchira, San Cristobal, Tachira, Venezuela; 4 Instituto de Microbiología y Bioquímica, Universidad Austral de Chile, Valdivia, Los Ríos, Chile; 5 Instituto de Producción Animal, Facultad de Ciencias Agrarias y Alimentarias, Universidad Austral de Chile, Valdivia, Los Ríos, Chile

**Keywords:** Solubilization of phosphates, *Penicillium rugulosum*, *Enterobacter cloacae*, Microbial co-inoculation

## Abstract

**Background:**

Forage production in tropical soils is primarily limited by nutrient deficiencies, especially nitrogen (N) and phosphorus (P). The use of phosphate rock by plants is limited by its low and slow P availability and microbial phosphate solubilization is the main mechanism for P bioavailability in the soil-root system. The objectives of this study were (i) select a nitrogen-fixing bacteria which could be used as a co-inoculant with the *Penicillium rugulosum* IR94MF1 phosphate-solubilizing fungus and (ii) evaluate under field conditions the effect of inoculation combined with phosphate rock (PR) application on yield and nutrient absorption of a *Urochloa decumbens* pasture which was previously established in a low-fertility, acidic soil.

**Methods:**

Various laboratory and greenhouse tests allowed for the selection of *Enterobacter cloacae* C17 as the co-inoculant bacteria with the IR94MF1 fungus. Later, under field conditions, a factorial, completely randomized block design was used to evaluate the inoculation with the IR94MF1 fungus, the IR94MF1+C17 co-inoculation, and a non-inoculated control. Two levels of fertilization with PR treatment (0 kg/ha and 200 kg/ha P_2_O_5_) were applied to each.

**Results:**

During five consecutive harvests it was observed that the addition of biofertilizers significantly increased (*p* < 0.05) the herbage mass and N and P assimilation compared to the non-inoculated control. However, no statistically significant differences were observed for the PR application as P source.

**Conclusion:**

*P. rugulosum* IR94MF1 is capable of solubilizing and accumulating P from the phosphate rock, making it available for plants growing in acid soils with low N content. These inoculants represent a good option as biofertilizers for tropical grasses already established in acidic soils with low N content.

## Introduction

Approximately 70% of the arable land in Venezuela has low pH values that limit agricultural productivity due to nutrient deficiencies (Artigas et al., 2019). Among these nutrients, nitrogen (N) and phosphorus (P) are two of the most important and limiting elements in the acidic soils of the tropics (FAO–IAEA, 2000). On the other hand, grasslands are the predominant ecosystem in the acidic soils of Venezuela and the grass *Urochloa decumbens* (Stapf.) R.D. Webster (syn *Brachiaria decumbens*) has been established for more than 50 years in livestock systems (Guenni, Seiter & Figueroa, 2008). Therefore, grass production on these soils is limited, leading to low phytomass productivity with poor nutritional quality for meat and milk production systems.

One of the most common practices to increase pasture productivity is the use of synthetic N and P-based fertilizers. However, this practice also increases production costs and provides only short-term effects in acidic soils (Casanova, 2007; Gurmessa, 2021). Furthermore, the fertilizer industry uses fossil fuels which generate greenhouse gas emissions, and an inappropriate use of fertilizers can contaminate soil and water. On the other hand, diverse soil microorganisms associated with plant roots can promote crop growth through different mechanisms, including biological N-fixation, phosphate solubilization, and the production of phytohormones (Jacoby et al., 2017; Sarmah & Kumar, 2022). Recently, the use of inoculants comprised of microorganisms as biofertilizers has demonstrated a great potential to mitigate the negative impacts of low soil fertility and abiotic stresses (Bargaz et al., 2018; Timmusk et al., 2017). Studies have shown that the use of biofertilizers can allow up to 50% reduction in the use of NPK fertilizers without resulting in adverse effects on crop growth, nutritional quality or yield (Thilagar, Bagyaraj & Rao, 2016). For example, fungal inoculants such as *Penicillium bilaii* and *P. janthinellun* have been commercially developed with success in different countries as phosphate-solubilizing biofertilizers and enhancers of P assimilation in sorghum, soy and corn (Monsanto BioAg, 2016), wheat (Harvey, Warren & Wakelin, 2009), rice, and grasses (Zambrano et al., 2015). The development of inoculants with various modes of action represents a sustainable resource for agricultural systems, decreasing the probability of pasture degradation, increasing nutrient absorption and contributing to increased carbon capture (Timmusk et al., 2017).

Recently, a new approach to “rhizosphere engineering” proposes the use of microbial co-inoculation or consortia in plants. These can effectively associate to the biological structural networks in native soils stimulating the recuperation of microbial functional groups linked to soil fertility (Ryan et al., 2009; Woo & Pepe, 2018). It is known that P availability often limits biological nitrogen fixation by diazotrophic bacteria. Also, P-solubilizing microorganisms can convert insoluble P into low molecular weight of organic and ionic forms available to plants (Li et al., 2020). In this sense, there are inoculants that integrate two or more microorganisms with different plant growth-promoting strategies, such as the biofertilizer BioGro® which was developed in Vietnam for rice crops and comprised the nitrogen-fixing bacteria *Pseudomonas fluorescens*, the cellulose/protein/starch-decomposing bacteria *Bacillus subtilis* and *B. amyloliquefaciens* (E19), and the inorganic phosphate solubilizer yeast, *Candida tropicalis* (HY) (Nguyen et al., 2014). Moreover, the inoculant Biovitis Céres®, among other products, is comprised of *P. fluorescens + Trichoderma harzianum* and can strengthen roots, improving nutrient assimilation and increasing resistance to abiotic stress in wheat (Woo et al., 2014).

The use of phosphate rock (PR) has been considered an important practice for P replacement in acidic soils and tropical pastures, primarily due to the scarce accessibility and high cost of soluble phosphorus fertilizers (Casanova, Salas & Toro, 2002; Weil, 2000). However, PR has a low and slow nutrient availability for the plants. For this reason, technologies must be developed to improve the efficiency of the application of natural recalcitrant phosphate resources as PR (Menezes-Blackburn et al., 2018). It is known that diverse microorganisms have the capacity to solubilize inorganic phosphates present in the rocks or phosphoric minerals (Antoun, 2012). Reyes et al. (1999b) reported that the fungus *P. rugulosum* strain IR94MF1 possesses highly functional solubilization mechanisms of the natural phosphoric rocks when using various modes of action. Thus, the development of biofertilizers comprised of microorganisms capable of solubilizing the mineral phosphate in the soil could be a promising alternative for the production of tropical pastures, especially in nutritionally limited soils.

We propose that co-inoculation (diazotrophic bacteria + *P. rugulosum*) in conjunction with the application of a low-available phosphorus source (PR) can increase phytomass production and N and P content of *U. decumbens* in a strongly acidic soil under field conditions. Thus, the objectives of this study were: (1) To select a nitrogen-fixing bacteria as a co-inoculant with the phosphate-solubilizing fungus *P. rugulosum* strain IR94MF1 which could exert an effect in the rhizosphere of *U. decumbens* and promote its growth, and (2) to evaluate under field conditions the combined effect of inoculation and PR application on both, *U. decumbens* yield and nutrient absorption, in an established pasture at an acidic soil with low-fertility and without the application of N and P fertilizers.

## Materials and Methods

### Origin of microbial isolates

The fungus studied was the IR94MF1 strain of *P. rugulosum*, an inorganic phosphate-solubilizing fungus for poorly soluble phosphate isolated from a natural outcropping of the PR mine of Monte Fresco located in the Venezuelan State of Táchira (Reyes et al., 1999a, 1999b), and nine bacterial strains with free-living diazotrophic characteristics (Table 1). Eight of these bacteria had previously been isolated from rhizospheres of *U. decumbens* and *U. humidicola* grasses collected from different agro-ecological zones and acidic soils. All bacterial isolates were characterized with various conventional biochemical tests to rule out any pathogenic potential (Table S1). The *Enterobacter cloacae* C17 previously isolated from the same phosphate mine soil was also used (Reyes, Valery & Valduz, 2006; Reyes & Antoun, 2010). Bacteria were selected on the basis of their ability to grow in a nitrogen-free culture medium and the absence of inhibition of germination and growth of sprouts and seedlings of different grasses (Barbosa, 2007) and other species (Reyes et al., 2008).

**Table 1:** Origin of the microorganisms selected in this study.

Microorganism	Isolate	Soil type	Soil pH^a^	Climate^b^	Isolated	Reference
*Azospirillum* spp.	A4	Entisols	4.4	Am	*Urochloa humidicola*	Used for the first time
*Azospirillum* spp.	A10		4.5		*U. decumbens*	
*Azospirillum* spp.	A11		4.5		*U. decumbens*	
*Azotobacter* spp.	C55	Inceptisols	5.2	Af	*U. decumbens*	
*Azospirillum* spp.	C5		5.2		*U. decumbens*	Reyes et al. (2008)
*Azospirillum* spp.	A5	Entisols	4.5	Am	*U. decumbens*	
*Azospirillum* spp.	A3	Inceptisols	5.6	Cwb	*U. decumbens*	
*Rhizobium radiobacter*	C11	Entisols	4.2	Am	*U. humidicola*	Reyes & Antoun (2010)
*Enterobacter cloacae*	C17	Ultisols	5.4	Cwb	*Iresine herbotit*	Reyes & Antoun (2010)
*Penicillium rugulosum*	IR94MF1		5.4		Apatite mine soil	Reyes et al. (1999a, 1999b)

**Note:**

^a^: Soil pH in water 1:2.5; Af: ^b^: Tropical rainforest; Am: tropical monsoon; Cwb: Temperate highland with dry winters.

### Preliminary selection of bacterial strains

#### Screening of phosphate solubilizing bacteria

The fungus *P. rugulosum* is considered as a phosphate solubilizing microorganism and its solubilization mechanism has been studied previously (Reyes et al., 1999a, 1999b, 2001; Reyes, Bernier & Antoun, 2002). For this reason, in this work no phosphate solubilization tests were carried out on the fungus. The phosphate-solubilizing activity of the nine bacteria was measured by the presence of dissolution halos or clear zones around the colonies according to Reyes, Valery & Valduz (2006). The inorganic phosphate solubilizing capacity in liquid media of *P. rugulosum* IR94MF1, *E. cloacae* C17 and *R. radiobacter* C11 was evaluated in a previous work (Z Valduz & A Valery, 2018, unpublished data). An *in vitro* test of the inorganic phosphate solubilizers was performed for the nine bacterial strains according to Reyes et al. (1999a). The agar cultivation media used were: Minimum medium, manita medium for *Azotobacter* and Congo red medium for *Azospirillum*. For each cultivation medium, the source of soluble phosphate was replaced by low solubility P sources: Hydroxyapatita [Ca_5_(PO_4_)_3_OH 2.7 g/L]; aluminum phosphate (AlPO_4_ 1.97 g/L) and iron phosphate (FePO_4_ 4.86 g/L). In each medium, with and without the modification of the P source, 5 μl of inoculant at a concentration of 1 × 10^8^ CFU/ml was cultivated in triplicate. On the fifth day of incubation at 28 °C, the diameter of the bacterial colony and the halo of solubilization were measured using a micrometer.

#### *U. decumbens* seed germination

The effect of the nine bacterial strains and the IR94MF1 fungus on the germination of *U. decumbens* seeds was evaluated in laboratory conditions. One thousand seeds of *U. decumbens* were disinfected with 80% ethanol and agitated for 8 min. Seeds were passed through a commercial solution of 5.25% sodium hypochlorite for 30 min and then washed with sterile, distilled water. The bacterial strains were multiplied using the solid media for *Azotobacter* and *Azospirillum* while the complete media was used for the fungus (Reyes et al., 1999a). This was achieved using a suspension of each strain in a 0.89% saline solution. The microbial concentration for the fungus was brought to 3 × 10^8^ and for the bacterial strains to 7 × 10^9^ CFU/ml using a Neubauer chamber for their respective counts. Seeds were placed directly in the chamber in Petri dishes in a water agar medium as in Reyes, Bernier & Antoun (2002). The control treatment consisted of treated seeds without microbial inoculation. The entire process was performed in aseptic conditions within a laminar flow chamber (LABCONCO CLASS II). Seeds were incubated in darkness at 27 °C for 96 h and later placed in a growth chamber Biotronette® Mark III model Lab-Line 846 and 12 h of light to count the total number of germinated seeds with emerged radicle and plumule after 21 days.

### Experiments

#### Greenhouse trial

Six microbial inoculants were selected after the phosphate solubility and *U. decumbens* seed germination trials were completed. The greenhouse treatments were: *P. rugulosum* IR94MF1 fungus, five fungus-bacteria co-inoculants and a non-inoculated control. The production of isolates in liquid media for later inoculation was performed according to Reyes, Valery & Valduz (2006), and the inoculation of the seeds was done as previously described. In a microcosm trial, the experimental unit was a pot with 4.5 kg of dry and un-sieved soil. The soil was a sandy-loam, had 1.12% organic matter (Walkley-Black), a pH of 4.45 (in water 1:2.5), 6 mg/kg P (Bray-Kurtz I), 30 mg/kg of K, 151 mg/kg of Ca, 22 mg/kg of Mg, and 0.02 dSm/cm. Fifteen inoculated seeds were sown in each pot and thinned after 10 days to leave four grass seedlings per pot. To guarantee the presence of the inoculants, at the start and finish of each harvest 30 mL of a new microbial suspension was injected into the soil at a depth of 2.5 cm beneath the soil surface.

The study was established using a factorial, completely randomized block design with seven repetitions per treatment. The 14 treatments consisted of seven inoculants including a control and two levels of phosphate fertilization: A control dose (0 kg P_2_O_5_/ha) and a dose of 400 kg P_2_O_5_/ha, using the PR of Monte Fresco. The PR had been previously ground and sieved at 100 mesh (27% P_2_O_5_ total, 30% Ca) reported as a PR of low solubility in citric acid and 2% formic acid (Pérez, Smyth & Israel, 2007). Neither N nor P was applied while all treatments received the same dose of micronutrients, which consisted of a fourth of the concentration of the nutritive Hoagland solution.

The study was performed in a greenhouse with an average temperature of 26 ± 4 °C and 75% relative humidity. Two harvests were performed, one at 45 days and one at 75 days after planting. Grasses were harvested at 5 cm residual height. In the second harvest, the dry weight of roots greater than 2 mm in diameter was measured.

#### Field trial

The study was conducted at the university experimental station, “Hacienda Santa Rosa” (municipality Fernández Feo, Táchira, Venezuela 7°33′53″ N; 72°02′13″ W, 330 m.a.s.l) from October 2007 through April 2008. The soil at the site was a Typic Ustorthents with a sandy-loam texture, 1.07% organic matter (Walkley-Black), pH 4.82 (in water 1:2.5); 6.0 mg/kg P (Bray-Kurtz I), 37 mg/kg K, 228 mg/kg of Ca, 19 mg/kg Mg and 0.05 dSm/cm. According to Köppen, the study site has a tropical monsoon climate (Am) with a unimodal precipitation distribution. Monthly accumulated precipitation (mm) and average, minimum and maximum monthly temperatures (°C) during the study were registered at the Venezuelan Airforce’s Santo Domingo station (Table 2).

**Table 2:** Monthly accumulated rainfall and monthly and mean, minimum and maximum temperatures at the field trial from October 2007 to April 2008 (data from the weather station at Santo Domingo-FAV).

Harvests^a^	Date	Monthly accumulated rainfall (mm)	Monthly daily temperature (°C)		
			Mean	Minimum	Maximum
1^st^	Oct-07	328.8	24.8	20	30
	Nov-07	234.5	24.8	21	30
2^nd^	Dec-07	132.8	24.0	21	31
3^rd^	Jan-08	11.5	23.4	21	30
	Feb-08	150.0	24.1	21	29
4^th^	Mar-08	97.4	24.1	20	30
5^th^	Abr-08	435.8	25.8	21	31

**Note:**

a Harvests carried out during the field trial.

The bacterial strain *E. cloacae* C17 used as a co-inoculant with IR94MF1 fungus was determined based on the results of the greenhouse and laboratory trials. The experiment was performed in a *U. decumbens* pasture that had been established three years earlier and had not been fertilized during that time. A factorial, completely randomized block design with three repetitions was used and each experimental plot was 64 m^2^ (8.0 m × 8.0 m).

Three inoculation treatments were evaluated: The IR94MR1 fungus, the IR94MF1 + *E. cloacae* C17 co-inoculation and a non-inoculated control. The second factor was the PR dose, which had two levels; 0 kg PR and 750 kg PR/ha equivalent to 0 kg/ha and 200 kg/ha P_2_O_5_, respectively. The six treatments were applied to each plot 3 days after cutting the pastures to a uniform residual height of 10 cm. The inoculants were applied directly to the soil at the base of the tillers. The dose of the IR94MF1 was 1.5 L/ha at a concentration of 3 × 10^8^ CFU/mL, while the dose of the bacteria was 500 mL/ha at a concentration of 6 × 10^9^ CFU/mL. The PR was applied according to the recommendations for grasses in acidic soils with the previously described fertility characteristics (Casanova, 2007). Neither N nor K was applied, and no pH correction was performed. Five consecutive harvests at 10 cm residual height were performed when the plant canopy reached 30 cm in height. The resting days between harvest events were as follows: first harvest (28 days), second harvest (30 days), third harvest (35 days), fourth harvest (35 days) and fifth harvest (30 days) (Table 2). At each harvest, the live herbage mass and growth rate were determined, and the estimation of annual live herbage mass and the annual assimilation of N and P were estimated with the sum of all the harvests performed during the experimental period.

### Analysis of plant material

In both experiments, the grass’ dry weight (leaf + stem) was determined by drying the samples in a forced air oven at 65 °C for 72 h. Leaves and stems were then grounded so that the N content was determined using the Kjeldahl method and the P content was determined by colorimetry (Temminghoff & Houba, 2004).

### Statistical analysis

Results of the phosphate solubilization were presented with descriptive and nominal statistics. The assumptions of normality and homogeneity of the residuals of the data were evaluated through the Shapiro-Wilk test. The greenhouse germination trial data and the variables from the field trial (accumulated herbage mass, N and P accumulated assimilation) were analyzed using an ANOVA according to their respective designs. The two cuttings of the greenhouse trial were analyzed as cumulative data for aerial dry weight, root dry weight, N and P assimilated. Variables measured in the field trial were analyzed focusing on mixed linear models with repeated measurements using the command GENLINMIXED in IBM SPSS version 25. The model included the block as a random effect and the harvest, inoculants and P application as fixed effects. Repeated measurements over time within the blocks were modeled using the autoregressive error (AR(1)) structure and the Kenward-Roger process was used to approximate the degrees of freedom. The Akaike’s information criteria was used to select the best covariance structure. Means were compared using Fisher’s least significant difference (LSD) test with significance *p* < 0.05 for all procedures.

## Results

### Detection of phosphate solubilizing isolates

This test made it possible to select strains of diazotrophic bacteria capable of growing and solubilizing poorly soluble inorganic phosphates. The isolates A5, C5, C55, *R. radiobacter* (C11) and *E. cloacae* (C17) presented >0.5 mm of growth on all culture media containing hydroxyapatite, iron phosphate and aluminum phosphate (Table 3).

**Table 3:** Colony growth (mm) and solubilization halo formation of bacteria in various solid culture media with low available phosphate source.

Isolate	Minimum mineral medium			Manita medium			Congo red medium		
	HA	AlPO_4_	FePO_4_	HA	AlPO_4_	FePO_4_	HA	AlPO_4_	FePO_4_
	Diameter of bacterial colony (mm)								
*Azospirillum* spp. A4	ncg	1.0 ± 0.2	ncg	ncg	ncg	ncg	2.0 ± 0.3	2.1 ± 0.5	0.5 ± 0.2
*Azospirillum* spp. A10	1.3 ± 0.2	1.1 ± 0.3	ncg	1.0 ± 0.2	0.7 ± 0.2	ncg	2.0 ± 0.3	1.5 ± 0.4	1.2 ± 0.2
*Azospirillum* spp. A11	1.2 ± 0.2	1.7 ± 0.3	ncg	ncg	ncg	1.0 ± 0.2	2.0 ± 0.4	2.2 ± 0.3	1.0 ± 0.3
*Azotobacter* spp. C55	1.5 ± 0.3	1.8 ± 0.3	1.0 ± 0.2	0.7 ± 0.2	1.0 ± 0.2	0.7 ± 0.2	3.0 ± 0.5	4.2 ± 0.5	2.1 ± 0.4
*Azospirillum* spp. C5	1.5 ± 0.2	1.1 ± 0.2	1.1 ± 0.2	1.5 ± 0.2	1.2 ± 0.2	0.5 ± 0.2	3.8 ± 0.5	3.9 ± 0.6	2.8 ± 0.6
*Azospirillum* spp. A5	1.5 ± 0.3	1.2 ± 0.3	1.0 ± 0.3	0.5 ± 0.2	2.0 ± 0.3	1.2 ± 0.2	1.5 ± 0.3	2.0 ± 0.4	2.8 ± 0.5
*Azospirillum* spp. A3	2.5 ± 0.5	1.5 ± 0.2	ncg	ncg	ncg	0.8 ± 0.2	2.5 ± 0.5	3.0 ± 0.6	2.5 ± 0.4
*R. radiobacter* C11	2.3 ± 0.4 + b	2.3 ± 0.3	1.5 ± 0.2	2.1 ± 0.3	2.1 ± 0.3	2.1 ± 0.2	4.1 ± 0.6	3.7 ± 0.7	3.1 ± 0.5
*E. cloacae* C17	2.1 ± 0.4 + a	1.7 ± 0.3	1.2 ± 0.2	2.7 ± 0.3 + b	1.5 ± 0.3	0.6 ± 0.2	3.0 ± 0.6	3.2 ± 0.5	3.2 ± 0.7

**Note:**

HA, hydroxyapatite; AlPO_4_, aluminium phosphate; FePO_4_, iron (III) phosphate; ncg, no colony growth; +a: halo with radius between 0.5 and 1.1 mm beyond the edge of the colony; +b: halo with radius between 1.1 and 1.8 beyond the colony edge. Values represent the mean ± standard error of the mean.

**Figure 1:**
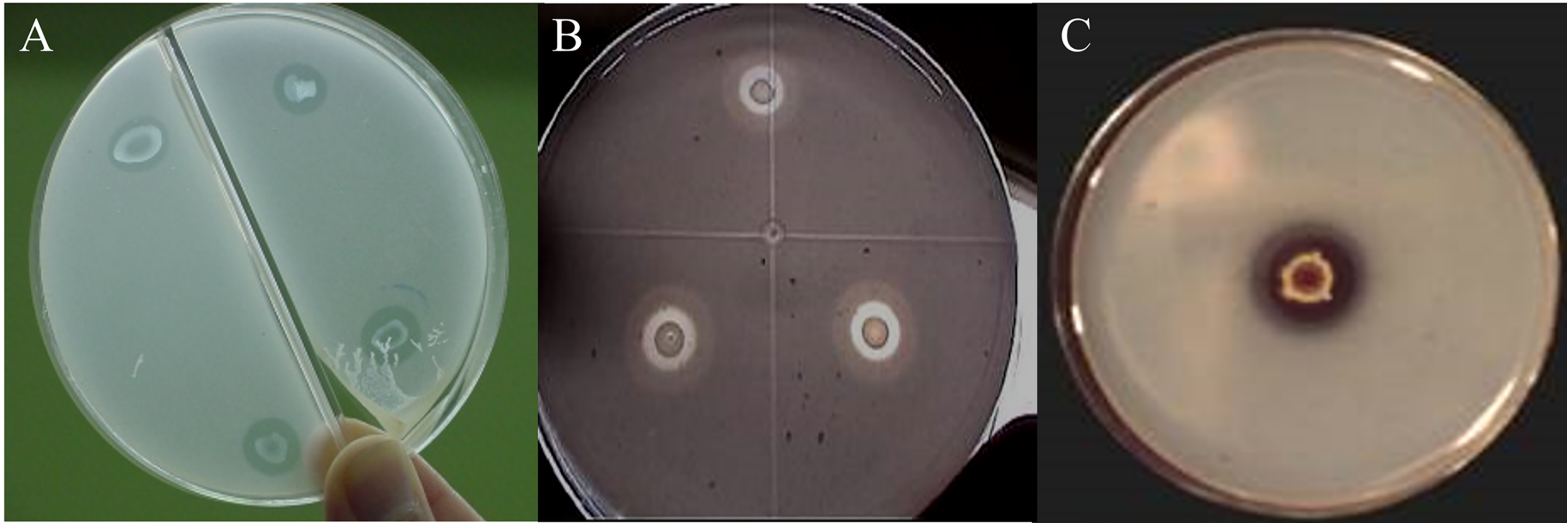
Phosphate solubilization halos in a minimal medium with hydroxyapatite. (A) *Enterobacter cloacae* C17. (B) *Rhizobium radiobacter* C11. (C) *Penicillium rugulosum* IR94MF1.

Of the nine strains, *R. radiobacter* C11 and *E. cloacae* C17 showed the formation of solubilization halos in culture media containing hydroxyapatite, with a radius between 0.5 and 1.8 mm measured from the edge of the colony (Fig. 1). However, only the *E. cloacae* C17 showed Ca phosphate solubilization halo in minimal mineral and mannite culture media. None of the strains presented transparent halos surrounding the colonies in aluminum phosphate and iron phosphate media.

### Growth-promoting effect on seed germination of *U. decumbens*

**Figure 2:**
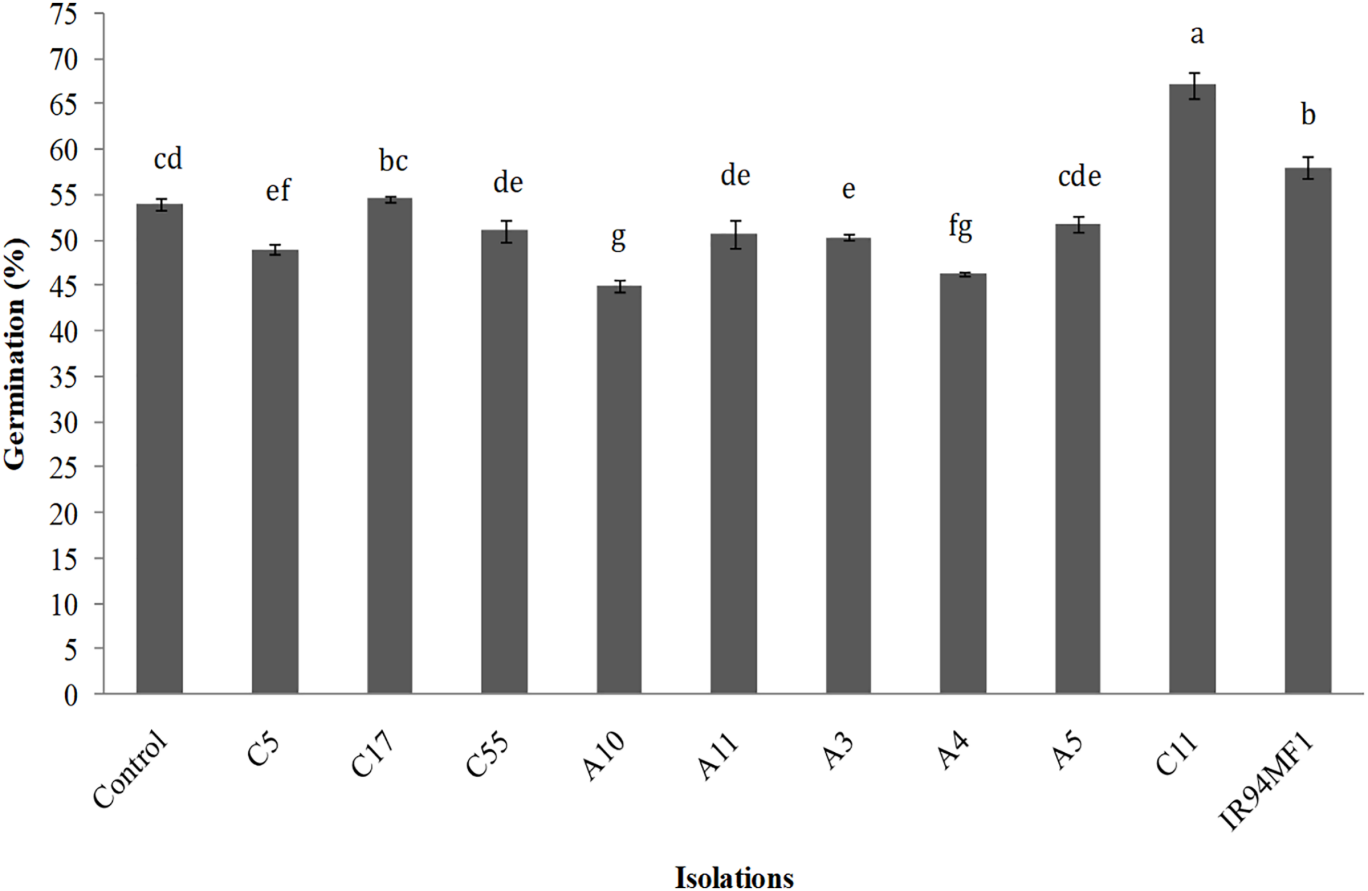
Germination of *U. decumbens* inoculated with free living N-fixing bacteria and the phosphate solubilizing fungus *P. rugulosum* (IR94MF1). Isolates A3, A4, A5, A10, A11 and C5 are *Azospirillum* spp.; C55: *Azotobacter* spp.; C11: [i]Rhiz.

Inoculation had a significant (*p* < 0.05) but variable effect on the germination of *U. decumbens* seeds. The *R. radiobacter* C11 isolate presented the greatest germination value (67%), while the IR94MF1 fungus and the *E. cloacae* C17 bacteria showed similar values to those of the non-inoculated control (54%) (Fig. 2). However, the A10, A4 and C5 strains significantly decreased germination (*p* < 0.05).

### *E. cloacae* C17 as a potential co-inoculum of *P. rugulosum*

The focus of this work was to select a non-antagonistic diazotrophic bacteria with the ability to have a synergistic effect with the solubilizing fungus P. *rugulosum* IR94MF1 in conjunction with the application of natural PR. Table 4 presents the significant effects of the inoculant’s application on the growth of *U. decumbens* in all studied variables. No increase in cumulative aerial dry weight (ADW) of inoculated plants compared to the control was observed (Table 4). The application of PR did result in differences in ADW of the *U. decumbens* seedlings under these conditions. The combination of the inoculation and PR application affected the root dry weight (RDW). The co-inoculant *P. rugulosum* + *E. cloacae* C17 without the application of P_2_O_5_ increased *U. decumbens* RDW by 64.61% while the 400 kg dose of P_2_O_5_ decreased RDW by 17.72%. However, the inoculation with *P. rugulosum* IR94MF1 and *P*. *rugulosum* + *R. radiobacter* C11 with the application of 400 kg P_2_O_5_ increased RDW compared to the control (0 kg P_2_O_5_). Similarly, the co-inoculant *P. rugulosum* + *E. cloacae* C17 presented higher N assimilation (38.55 mg N pot^−1^) and P assimilation (12.31 mg pot^−1^) compared to the non-inoculated control without PR. This evaluation allowed the diazotrophic bacterium *E. cloacae* C17 to be selected as a co-inoculum of *P. rugulosum* IR94MF1 to promote growth and N and P assimilation of *U. decumbens*.

**Table 4:** Effect of the application of microbial inoculants and phosphate rock (PR) on the accumulated aerial dry weight (ADW g/pot), root dry weight (RDW g/pot) and N and P uptake (mg/pot) of *U. decumbens* under greenhouse conditions.

PR application	Inoculant	ADW	RDW	N uptake	P uptake
		(g/pot)		(mg/pot)	
0 kg P_2_O_5_	No inoculant	4.23 ± 0.16 ab	3.56 ± 0.24 d	28.39 ± 1.50 c	10.99 ± 0.50 bc
	*P. rugulosum* IR94MF1	4.45 ± 0.20 a	4.35 ± 0.25 abc	33.32 ± 2.07 b	11.40 ± 0.44 ab
	*P. rugulosum* + *Azospirillum* spp. A11	3.64 ± 0.08 cd	3.89 ± 0.37 abc	26.54 ± 0.73 cd	9.91 ± 0.24 cd
	*P. rugulosum* + *Azospirillum* spp. C5	3.57 ± 0.10 cd	4.44 ± 0.25 bc	25.18 ± 0.40 cd	9.72 ± 0.33 d
	*P. rugulosum* + *Azotobacter* spp. C55	3.96 ± 0.10 bc	4.63 ± 0.14 b	27.12 ± 1.70 cd	11.39 ± 0.30 bc
	*P. rugulosum* + *R. radiobacter* C11	3.23 ± 0.22 d	3.62 ± 0.22 cd	22.52 ± 2.34 d	10.26 ± 0.60 bcd
	*P. rugulosum* + *E. cloacae* C17	4.58 ± 0.22 a	5.86 ± 0.48 a	38.55 ± 2.26 a	12.31 ± 0.45 a
400 kg P_2_O_5_	No inoculant	4.45 ± 0.20 a	3.50 ± 0.17 efg	30.26 ± 2.04 bc	11.61 ± 0.44 ab
	*P. rugulosum* IR94MF1	4.46 ± 0.11 a	4.48 ± 0.17 a	33.84 ± 0.94 ab	11.34 ± 0.37 abc
	*P. rugulosum* + *Azospirillum* spp. A11	3.45 ± 0.09 b	3.46 ± 0.25 c	22.79 ± 1.57 d	9.95 ± 0.41 d
	*P. rugulosum* + *Azospirillum* spp. C5	3.71 ± 0.11 b	3.26 ± 0.13 c	26.21 ± 1.04 cd	9.79 ± 0.21 d
	*P. rugulosum* + *Azotobacter* spp. C55	3.50 ± 0.13 b	3.59 ± 0.43 bc	23.46 ± 1.70 d	10.00 ± 0.37 cd
	*P. rugulosum* + *R. radiobacter* C11	3.72 ± 0.20 b	4.36 ± 0.21 ab	27.37 ± 1.67 cd	10.74 ± 0.61 bcd
	*P. rugulosum* + *E. cloacae* C17	4.42 ± 0.23 a	4.12 ± 0.54 abc	37.68 ± 2.86 a	12.25 ± 0.70 a
*p* value	Inoculant	<0.001	<0.001	<0.001	<0.001
	PR application	0.936	<0.001	0.998	0.868
	Interaction	0.077	<0.001	0.179	0.383

**Note:**

Values represent the mean ± standard error of the mean. Different letters within each column denote significant differences according to the LSD test at *p* < 0.05.

### Effect of *P. rugulosum* IR94MF1 and the consortium *P. rugulosum* + *E. cloacae* C17 on grassland of *U. decumbens*

In this work, no effect was observed by the application of inoculant and PR on the variables measured in the field (*p* > 0.05), but an effect was found across the five cuts performed. Thus, it was demonstrated that the application of the inoculants *P*. *rugulosum* IR94MF1 and the *P. rugulosum* + *E. clocae* C17 consortium during the harvests did not show an effect on dry matter yield and growth rate but did affect N and P assimilation (*p* < 0.001) (Tables 5 and 6). Likewise, it was found that the inoculants *P. rugulosum* IR94MF1 and *P. rugulosum* + *E. clocae* C17, independently of the dose of PR applied during the 4th cutting, increased N assimilation (*p* < 0.001) after the period of low rainfall recorded in the trial, when compared to the non-inoculated control (Table 6). Although, there were variable responses across cuts which could limit the consistency of the inoculants applied under field conditions with or without RP, it was found that the addition of inoculants increased cumulative forage mass yield by 17–33%, annual N assimilation by 21–38%, and P assimilation by 18–47%, compared to the non-inoculated control (Table 7). However, no differences were observed between the inoculants *P. rugulosum* IR94MF1 and *P. rugulosum* + *E. clocae* C17, nor was any difference found with the application of PR as a source of P in the variables analyzed.

**Table 5:** Effect of the application of phosphate rock (PR) and plant growth promoting microorganisms on the live aerial herbage mass (kg DM/ha) and growth rate (kg DM/ha/d) of *U. decumbens* in five successive harvests from November 2007 to April 2008.

PR application	Inoculant	Yield (kg DM/ha)				
		1^st^	2^nd^	3^rd^	4^th^	5^th^
0 kg P_2_O_5_	No Inoculant	1,234 ± 114.4 a	1,864 ± 199.9 a	841 ± 110.6 a	912 ± 128.4 b	1,455 ± 101.7 b
	*P. rugulosum* IR94MF1	1,349 ± 76.8 a	2,014 ± 135.7 a	866 ± 44.8 a	1,383 ± 117.4 a	1,738 ± 186.0 ab
	*P. rugulosum + E. cloacae* C17	1,155 ± 166.1 a	2,195 ± 76.5 a	752 ± 66.7 a	1,560 ± 202.9 a	2,048 ± 192.0 a
200 kg P_2_O_5_	No Inoculant	1,107 ± 145.1 a	1,346 ± 64.9 a	709 ± 65.2 a	1,115 ± 93.7 c	1,743 ± 175.1 a
	*P. rugulosum* IR94MF1	1,784 ± 357.3 a	1,418 ± 151.0 a	787 ± 32.3 a	1,975 ± 78.9 a	2,021 ± 144.8 a
	*P. rugulosum + E. cloacae* C17	1,424 ± 217.3 a	1,400 ± 101.3 a	895 ± 86.0 a	1,557 ± 179.9 b	1,937 ± 225.3 a

		Growth rate (kg DM ha/day)				
0 kg P_2_O_5_	No Inoculant	44.1 ± 4.09 a	62.1 ± 6.66 a	24.0 ± 3.16 a	26.1 ± 3.67 b	48.5 ± 3.39 b
	*P. rugulosum* IR94MF1	48.2 ± 2.74 a	67.1 ± 4.52 a	24.7 ± 1.28 a	39.5 ± 3.35 a	57.9 ± 6.20 ab
	*P. rugulosum + E. cloacae* C17	41.2 ± 5.93 a	73.2 ± 2.55 a	21.5 ± 1.90 a	44.6 ± 5.80 a	68.3 ± 6.40 a
200 kg P_2_O_5_	No Inoculant	39.5 ± 5.18 a	44.9 ± 2.16 a	20.3 ± 1.86 a	31.9 ± 2.68 c	58.1 ± 5.84 a
	*P. rugulosum* IR94MF1	63.7 ± 12.76 a	47.3 ± 5.03 a	22.5 ± 0.92 a	56.4 ± 2.25 a	67.4 ± 4.83 a
	*P. rugulosum + E. cloacae* C17	50.9 ± 7.76 a	46.7 ± 3.38 a	25.6 ± 2.46 a	44.5 ± 5.14 b	64.6 ± 7.51 a

**Note:**

The values represent the means ± standard error of the mean. Different letters within each column denote significant differences according to the LSD test at *p* < 0.05. Harvest dates: Nov-07; Dec-07; Jan-08 and Feb-08.

**Table 6:** Effect of the application of phosphate rock (PR) and plant growth promoting microorganisms on the assimilation of N and P in the aerial biomass of *U. decumbens* at five successive harvests.

PR application	Inoculant	N uptake (kg/ha)				
		1^st^	2^nd^	3^rd^	4^th^	5^th^
0 kg P_2_O_5_	No Inoculant	9.54 ± 0.92 a	9.77 ± 1.27 b	6.90 ± 1.13 ab	5.02 ± 0.72 b	11.81 ± 1.08 b
	*P. rugulosum* IR94MF1	11.04 ± 1.59 a	10.43 ± 0.82 b	7.95 ± 0.91 a	10.02 ± 1.66 a	12.88 ± 0.96 b
	*P. rugulosum + E. cloacae* C17	10.36 ± 1.67 a	14.15 ± 0.90 a	5.01 ± 0.52 b	9.95 ± 1.34 a	20.17 ± 1.97 a
200 kg P_2_O_5_	No Inoculant	9.54 ± 1.19 a	8.73 ± 0.91 a	6.67 ± 0.95 a	5.40 ± 0.72 c	13.85 ± 1.32 a
	*P. rugulosum* IR94MF1	15.10 ± 3.37 a	8.68 ± 1.20 a	6.84 ± 0.71 a	14.15 ± 1.38 a	15.16 ± 1.06 a
	*P. rugulosum + E. cloacae* C17	11.27 ± 1.96 a	9.18 ± 1.23 a	8.18 ± 0.79 a	10.20 ± 1.20 b	15.23 ± 1.56 a

		**P uptake (kg/ha)**				
0 kg P_2_O_5_	No Inoculant	3.66 ± 0.39 a	5.17 ± 0.52 b	2.57 ± 0.31 a	3.17 ± 0.35 a	5.10 ± 0.33 b
	*P. rugulosum* IR94MF1	4.37 ± 0.33 a	5.56 ± 0.50 b	2.26 ± 0.19 a	4.76 ± 0.58 a	6.65 ± 0.82 ab
	*P. rugulosum + E. cloacae* C17	4.03 ± 0.57 a	8.16 ± 0.30 a	2.65 ± 0.23 a	3.83 ± 0.93 a	7.37 ± 0.69 a
200 kg P_2_O_5_	No Inoculant	3.98 ± 0.47 a	4.35 ± 0.37 a	2.52 ± 1.86 b	2.83 ± 0.49 c	6.25 ± 1.04 a
	*P. rugulosum* IR94MF1	6.04 ± 1.22 a	3.83 ± 0.48 a	3.09 ± 0.25 ab	8.04 ± 0.91 a	8.42 ± 0.55 a
	*P. rugulosum + E. cloacae* C17	4.68 ± 0.99 a	4.22 ± 0.42 a	3.83 ± 0.54 a	5.94 ± 0.58 b	8.42 ± 1.34 a

**Note:**

Values represent the mean ± standard error of the mean. Different letters within each column denote significant differences according to the LSD test at *p* < 0.05.

**Table 7:** Effect of the application of phosphate rock (PR) and plant growth promoting microorganisms on the cummulative live aerial herbage mass production (kg DM/ha*year), N uptake (kg/ha*year) and P uptake (kg/ha*year) of *U. decumbens* in five successive harves.

PR application	Inoculant	Annual yield DM	N-uptake	P-uptake
			kg/ha/año	
0 kg P_2_O_5_	No Inoculant	6,306 ± 268.9 b	43.03 ± 1.55 b	19.67 ± 0.74 b
	*P. rugulosum* IR94MF1	7,350 ± 238.4 a	52.32 ± 1.86 a	23.30 ± 1.27 ab
	*P. rugulosum + E. cloacae* C17	7,709 ± 402.0 a	59.64 ± 4.15 a	26.05 ± 2.02 a
200 kg P_2_O_5_	No Inoculant	6,020 ± 240.9 b	44.20 ± 1.88 b	19.93 ± 0.74 b
	*P. rugulosum* IR94MF1	7,987 ± 390.2 a	59.93 ± 4.53 a	29.42 ± 1.70 a
	*P. rugulosum + E. cloacae* C17	7,214 ± 277.1 a	54.06 ± 1.66 a	27.12 ± 1.66 a
*p* value	Inoculant	<0.001	<0.001	<0.001
	PR application	0.851	0.654	0.054
	Interaction	0.169	0.089	0.136

**Note:**

Values represent the mean ± standard error of the mean. Different letters within each column denote significant differences according to the LSD test at *p* < 0.05.

## Discussion

The rhizopheric strains utilized in this study were derived from highly acidic soils with low P availability. Therefore, in a natural soil with high concentrations of relatively insoluble P, one could expect to find a high rate of phosphate solubilization by the microbial communities metabolically adapted to obtain P from sparingly soluble sources (Reyes, Valery & Valduz, 2006). In this way, among the bacterial isolates evaluated, *E. cloacae* C17 and *R. radiobacter* C11, presented hidroxiapatite solubilization and growth in the culture media with iron phosphates and aluminum phosphates without the visible observation of a solubility halo, which could be interpreted as low phosphate solubility. Various authors have reported that soil bacteria of the genera *Enterobacter* and *Rhizobium* are considered powerful phosphate-solubilizers (Chakraborty et al., 2019; Sharon et al., 2016). Similarly, Sharon et al. (2016) reported that *Enterobacter* sp. and *E. cloacae* strain 34,977 showed low solubilization rates in culture media with iron phosphate and aluminum phosphate. Probably, these enterobacteriaceae solubilize phosphates either by the extrusion of protons associated with ammonium assimilation or for the extracellular oxidation of glucose to gluconic acid and 2-cetoglunoic acid (Goldstein & Krishnaraj, 2007) or by the production of citric, oxalic, lactic/succinic and propionic acids, reported in diazotrophic and P-solubilizing bacteria isolated from tropical soils (Marra et al., 2012). The evaluation of phosphate solubility *in vitro* is a good indicator for the detection of microorganisms with potential as growth-promoters in plants, as is the case of the free-living diazotrophic bacteria *E. cloacae* C17 which presented potential to solubilize calcium phosphate, and the ability to show growth on media containing iron and aluminum phosphates. Although, different low solubility phosphates were used as a strategy in P dissolution, the production of a halo on a solid agar medium should not be considered as the only test for P solubilization. Thus, additional tests such as P dissolution tests in liquid medium and production of organic acids are necessary to be more rigorous in future inoculant selections (Bashan, Kamnev & de-Bashan, 2013). However, the effectiveness of these microorganisms will always be conditional on their relationship with plant roots and their environment.

Another variable evaluated was the effect of bacteria on the germination of *U. decumbens* seeds. The main objective of the germination test was to detect microorganisms capable of manifesting a relevant inhibitory, neutral or synergistic effect on *U. decumbens* seeds, as a tool for the selection of potential plant growth promoting microorganisms. Although no analyses were performed on the production of growth hormones by microorganisms, it is known that some bacteria of the genera evaluated in this work can produce inducing substances that promote or inhibit the germination process. For example, Humphry et al. (2007) on barley, Gholami, Shahsavani & Nezarat (2009) on maize, and Fahsi et al. (2021) on *Ziziphus lotus*, reported increases in seed germination with rhizobacteria of the genera *R. radiobacter* 204, *Azospirillum* sp., *Pseudomonas* sp. and *Bacillus cereus* J156, respectively. In this way, the same authors used the evaluation of germination of rhizobacteria-inoculated seeds as a technique to reveal future biofertilisers. However, an increase in seed germination as observed with *R. radiobacter* C11 on *U. decumbens* can often be an unfavorable indicator for the selection of a potential inoculant. In studies with inoculants, it is common to observe inconsistent performance in the measured variables (Richardson & Simpson, 2011), which has been attributed to different factors, such as survival and competitiveness of the inoculants introduced to the soil, the soil properties, the plant variety and its physiological state, and the limited understanding of the mechanisms which intervene in growth promotion (Kumar et al., 2017; Ryan et al., 2009). Unlike in the germination test, the bacterium *R. radiobacter* C11 did not increase the values of the variables measured in greenhouse conditions, in contrast to *E. cloacae* C17, which maintained its positive effect. In this case, the germination promotion was not a determinant variable but was complementary for the selection of the inoculant. It is also well-known that in unsterilized soils, the high competition from the native flora and predation by protozoa and nematodes causes the rapid, exponential decline of the introduced microbial populations until they reach an equilibrium with its environment (Martínez-Viveros et al., 2010). Therefore, the double inoculation in the greenhouse study assured the presence of the inoculants introduced into the rhizosphere of *U. decumbens* plants, ensuring their interaction with the plant and decreasing the inconsistencies which have been reported in the use of introduced microorganisms (Jacoby et al., 2017).

Inoculation with the co-inoculant *P. rugulosum* + *E. cloacae* C17 did not increase the aerial dry weight (ADW) of *U. decumbens*. However, it did increase the average values for N and P assimilation. This could be attributed to both the N-fixing capacity of the bacteria and the phosphate-solubilizing capacity of the fungus and the bacteria together. Additionally, it could also be due to the synergistic effect of this co-inoculant with the culture, derived from the increased capacity to absorb nutrients from the soil. Inagaki et al. (2015) reported similar results for inoculation with diazotrophic bacteria in acidic, sandy soils, where they found increased N and P concentrations in the foliar tissue of corn. The inoculation with the fungus *P. rugulosum* IR94MF1 alone and the *P. rugulosum* + *E. cloacae* C17 co-inoculant with and without P application, respectively, increased the root dry weight of *U. decumbens*. This response is considered positive, as it could improve the efficiency of nutrient acquisition and water transport to the plant.

In relation to the fungus, it has been demonstrated that various isolates of *Penicillium* sp. interact with crop roots (Murali et al., 2016; Reyes, Bernier & Antoun, 2002), participating in growth promotion of plants through the stimulation of longitudinal growth and density development of roots, solubilization of phosphates and other indirect mechanisms, such as phytohormone production and amino acid deposition (Antoun, 2012; Harvey, Warren & Wakelin, 2009; Williams & de Vries, 2019). The positive effects associated with the co-inoculant *P. rugulosum* + *E. cloacae* C17 under greenhouse conditions allowed its selection for the field evaluation.

Although in the field study there was not a statically significant interaction between the application of PR and the inoculants for the measured variables of *U. decumbens* pasture, it is known that native and introduced soil microorganisms play a fundamental role in the mobilization and solubilization of phosphates in soil. The field study results showed that the inoculation with the fungus *P. rugulosum* IR94MF1 alone and the co-inoculant *P. rugulosum* + *E. cloacae* C17 improved growth and N and P assimilation in *U. decumbens*. Similar results have been reported for the co-inoculant application such as *Enterobacter* sp, + *Microbacterium arborescens* in wheat (Kumar et al., 2017) and for *Rhizobium meliloti* + *Penicillum bilaii* in alfalfa (Rice, Olsen & Leggett, 1994). However, the synergistic activity of the co-inoculation of *P. rugulosum* + *E. cloacae* C17 was not as expected. In this case, limited information is available on the effects of inoculation of the P-solubilizing fungus and diazotrophic bacteria.

The fate of microbial inoculants which are applied under field conditions depends upon biotic and abiotic factors, and their success is due to response capacity and interaction with different inoculated plants (Alori, Glick & Babalola, 2017). Therefore, contrary to laboratory results, under field conditions individual inoculation with *P. rugulosum* IR94MF1 had a better effect on *U. decumbens* growth than did the co-inoculant. It is possible that the C17 bacterial strain did not survive the complex interactions in the soil-rhizosphere microbiomes and was perhaps victim of predation by native microfauna and mesofauna, organisms which influence the regulation of biogeochemical processes of the nutrients. Predation by protists has been shown to impact the abundance of introduced bacteria and play an important role in N mineralization (Ambrosini, de Souza & Passaglia, 2015). A good inoculant should colonize the rhizosphere and have the potential to alter and resist intrinsic changes in the microbial composition. However, the microbial colonization in the rhizosphere depends on the recognition and response to chemical signals from the root exudates, as the plant is able to favor certain inoculants in particular conditions, such as phosphate solubilizers under nutritionally deficient conditions (Ambrosini, de Souza & Passaglia, 2015; Dakora & Phillips, 2002).

Approximately 30–80% of P available in the soil is immobilized in various organic forms that have reduced bioavailability for plants (Richardson & Simpson, 2011). Thus, the phosphate solubilized by the fungus *P. rugulosum* IR94MF1 could have been immobilized by the fungal mycelium and other microorganisms preventing their binding to iron and aluminum, common elements in acidic soils. This process of phosphate and other nutrients immobilization in the native and introduced microbial communities allows for their delayed accessibility in the rhizosphere through cellular lysis and subsequent mineralization which forms compounds such as ammonia, nitrates, phosphates and ions which *U. decumbens* can absorb. Reyes, Bernier & Antoun (2002) similarly reported that the use of *P. rugulosum* IR94MF1 allowed for an increase in bacterial communities in the rhizosphere of maize due to the effect of phosphate solubilization and the liberation of other compounds by the rhizospheric microflora. Also, depending on the carbon source, *P. rugulosum* IR94MF1 can mobilize the phosphate present in the PR and the soil through different modes of action such as the production of gluconic acid and citric acid and the extrusion of protons (Reyes, Bernier & Antoun, 2002). Likewise, the inoculant *P. bilaii*, commercially known as JumpStart® (Antoun, 2012; Monsanto BioAg, 2016) has been reported to have similar mechanisms for phosphate solubilization which increase growth and phosphate assimilation of different crops in soils with low P availability. It is known that early phosphate assimilation by the plant leads to improved root development, which in turn increases herbage mass yield (Dakora & Phillips, 2002). Although root growth was not measured in the field study, in the greenhouse trial the inoculation with *P. rugulosum* IR94MF1 did increase root dry weight both with and without PR application. An indirect response of the phosphate-solubizing inoculants is the growth and development of the roots. This attribute permits the improved water and nutrient absorption, which increases herbage mass production and promotes tolerance to environmental stresses such as drought (Leite et al., 2019; Williams & de Vries, 2019). In addition, some studies show that growth-promoting microorganisms can improve soil water retention through the production of an extracellular matrix containing oligosaccharides and polysaccharides (Rubin, van Groenigen & Hungate, 2017).

Finally, various studies report that the application of PR from the Monte Fresco mine in acidic soils on pastures of *Urochloa* sp. demonstrate positive effects after the first year (Casanova, 2007; Pérez, Smyth & Israel, 2007). The recalcitrant nature of PR has a residual effect over time in phosphate liberation (Casanova, Salas & Toro, 2002; Smalberger et al., 2006). Therefore, the microbial solubilization of phosphates is the principal biological mechanism to initiate and regulate the flow and exchange of solubilized phosphate from the PR in the plant-soil-root interface. This microbiological mechanism enables the pasture to increase its growth and nutrient assimilation, which results in increased herbage mass production when there is sufficient soil moisture.

## Conclusions

The purpose of this work was to find a diazotrophic bacteria capable to exert a synergistic effect with the phosphate solubilizing fungus *P. rugulosum* IR94MF1 in conjunction with the application of natural PR. The isolate *E. cloacae* C17 showed a dual capacity for N fixation and phosphate solubilization *in vitro* and in greenhouse tests and was therefore selected as a potential inoculant for a consortium with *P. rugulosum* IR94MF1. However, when this consortium was introduced into the soil of a *U. decumbens* pasture, its effect was not significant when compared to the application of *P. rugulosum* IR94MF1 alone.

On the other hand, the fungus *P. rugulosum* IR94MF1 inoculated on its own showed a beneficial effect on forage production, and N and P assimilation through the wide climatic and soil moisture conditions registered through the growing season. These results present the fungus *P. rugulosum* IR94MF1 as a good alternative for biofertilization in the sustainable management of *U. decumbens* pastures, since the fungus is a microorganism that mobilizes P from phosphate rocks and P fixed in acid soils with low N content. However, future agronomic studies are required to test the effect of the fungus with other tropical forage grasses under different soil and climatic conditions.

## Supplemental Information

10.7717/peerj.18610/supp-1 Supplemental Information 1 Conventional biochemical tests performed on bacterial isolates.

10.7717/peerj.18610/supp-2 Supplemental Information 2 Raw data.

## Additional Information and Declarations

### Data Availability

The following information was supplied regarding data availability: The raw data are available in the Supplemental File.
