# Supplementary material for: The use of phosphate rock and plant growth promoting microorganisms for the management of Urochloa decumbens (Stapf.) R.D. Webster in acidic soils
Source: PeerJ. 2024 Dec 6;12:e18610. doi: 10.7717/peerj.18610 (PMC11627078; doi:10.7717/peerj.18610)
Supplement: Supplemental Information 1 [file peerj-12-18610-s001.docx]

Table S1 **Conventional biochemical tests performed on bacterial isolates**

|  | **Bacterial isolates** | | | | | | | | |
| --- | --- | --- | --- | --- | --- | --- | --- | --- | --- |
| **Test** | *Azospirillum* spp. C5 | *Azotobacter* spp. C55 | *Azospirillum* spp. A10 | *Azospirillum* spp. A11 | *Azospirillum* spp. A4 | *Rhizobium radiobacter.* C11 | *Enterobacter cloacae.* C17 | *Azospirillum* spp. A5 | *Azospirillum* spp. A3 |
| Gram stain | - | - | - | - | - | - | - | - | - |
| Capsule | - | - | - | - | - | + | - | + | - |
| Motility | - | - | - | - | - | + | + | - | + |
| Growth in NaCl 2% | + | + | + | + | + | + | + | - | - |
| Growth in NaCl 3% | + | + | + | + | + | + | + | - | - |
| Growth in NaCl 5% | + | + | - | + | - | + | + | - | - |
| Hemolysis on blood agar | - | - | - | - | - | - | - | - | - |
| Catalase | - | - | + | + | + | + | - | - | + |
| Protease | - | + | - | - | - | - | - | - | - |
| Indole | - | - | - | - | - | - | - | - | - |
| Nitrate | - | - | - | - | - | - | - | + | + |
| Urease | - | - | - | - | - | - | - | + | + |
| Mannitol salt | - | - | - | - | - | - | - | + | + |
| Citrate | - | - | - | - | - | + | + | - | - |
| Starch | - | - | - | - | - | - | - | + | - |
| Sucrose | + | + | - | - | - | + | + | - | + |
| Dextrose | + | + | + | + | + | + | + | + | + |
| Maltose | + | + | - | - | - | - | - | - | - |
| Lactose | + | + | - | - | - | - | - | - | + |
| Mannitol | + | + | + | + | + | + | + | + | + |

Notes:

+: Presence of bacterial growth or positive test reaction

-: Absence of bacterial growth or negative reaction to the test
